# The Relationship between Clinic Visit Accompanied by Family and Dementia Severity in Taiwan

**DOI:** 10.3390/ijerph18041792

**Published:** 2021-02-12

**Authors:** Chih-Yen Chang, Shou-Jen Lan, Chiao-Lee Chu, Ching-Sung Ho

**Affiliations:** 1 Department of Medical Education and Research, Jen-Ai Hospital, Taichung 41256, Taiwan; tptzuyen@mail.jah.org.tw; 2 Department of Eldercare, Central Taiwan University of Science and Technology, Taichung 40601, Taiwan; 3 Department of Medical Research, China Medical University Hospital, China Medical University, Taichung 40447, Taiwan; sjlan@asia.edu.tw; 4 Department of Healthcare Administration, Asia University, 500, Lioufeng Rd., Wufeng, Taichung 41354, Taiwan; 5 Department of Long Term Care, National Quemoy University, 1 University RD, Jinning Township, Kinmen 89250, Taiwan; chiaoleechu@gmail.com

**Keywords:** dementia, CDR scores, educational level, family support, Taiwan

## Abstract

Introduction: This study analyzes the severity of dementia status with clinical dementia rating (CDR) score distribution among patients according to various family functional and sociodemographic issues. Methods: A cross-sectional study was performed in a regional hospital in Central Taiwan. The sample consisted of 318 patients who came to the clinic from May 2018 to April 2019, and who were diagnosed by the physicians with CDR scores ≧ 0.5. The Chi-Square test and binary logistic regression analyses were performed for inferential statistical analysis. Results: The mean age of the sample was 78.7 ± 8.51 years, and 61.6% of the samples’ CDR scores were equal or less than 1.0. Patients visiting the clinic were accompanied by spouses (21.7%), sons or daughters-in-law (40.6%), daughters (23.6%). Of the sample, 142 (44.3%) patients live with sons. Patients with a lower educational level had higher CDR scores. Compared to the patients who went to the clinic by themselves, the higher OR values of CDR scores ≧ 2 are found in patients who were accompanied by other relatives (OR = 18.871, 95% C.I. = 3.117–114.237, *p* = 0.001), or spouse (OR = 10.783, 95% C.I. = 1.996–58.245, *p* = 0.006). Conclusion: The family member who accompanied the patient to a clinic visit and the patient’s educational level are both significant issues relating to the severity of dementia.

## 1. Introduction

Dementia is a serious health problem worldwide, with cases rapidly increasing with the growth of the elderly population. It is estimated that 5.3 million people in the US have Alzheimer’s disease (AD) and it is predicted that 7.7 million people will be impacted by 2030 [1]. The number of dementia patients will double every 20 years to 42 million in 2020 and 81 million by 2040 [2]. In Taiwan, there has been a quick increase in the percentage of the population over the age of 65 from 6.8% in 1992 to 11.1% in 2012 [3]. The number of dementia patients among the elderly was about 130,000 people in 2012, increasing from 50,000 people in the early 1990s in Taiwan [4]. It is estimated that around a quarter of Taiwanese elderly people are identified as intellectual function impairment. The prevention and treatment of dementia has become a significant health policy issue in Taiwan.

Various risk factors make individuals prone to dementia. According to a nationwide cohort study in Taiwan, it is found that people with the disease of ischemic stroke, transient ischemic attack and cerebrovascular hemorrhage had a significantly greater risk of dementia afterward [5]. Community studies show that people with a lack of education or low education are more likely to have dementia or Alzheimer’s disease [6]. Gender difference also existed in the prevalence of dementia; it is found that more women than men have Alzheimer’s or other dementia-related health issues. The prevalence of women with Alzheimer’s is twice as high in men in the USA [7]. However, the effect of education and gender relating to the dementia status in Taiwan needs further investigation.

Informal caregivers usually are indispensable in dementia care, which is particularly time-intensive, and many caregivers are required to give up their usual work. Informal care in the USA was estimated to cost USD 18 billion annually in 1998 [8]. There are many research studies that discuss the psychological, physical, and economic burden as well as the stress of the caregivers [9,10,11]. It is estimated that 70% to 80% of the seniors in Taiwan are taken care of by their family relatives when they are sick or disabled, since filial piety is an important virtue in Taiwan society [12]. Early stage Alzheimer’s dementia patients are usually cared for at home, so the primary home caregivers are regarded as the persons who have most participated in the patient’s care and taken the lead on healthcare decision making. It is found that son and daughter-in-law pairs were the most common primary caregivers of patients with dementia in Korea [13], and the result may be due to Asia’s traditional culture. The eldest surviving son of the family is usually expected to take the duties of looking after his parents in Taiwanese culture, and a daughter is no longer considered as a member of her original family after getting married or leaving home [14]. Family members’ care in regular daily life is important for the diagnosis and treatment of dementia patients [15,16,17]. However, there is limited knowledge of which family caregiver attributes are valued or helpful to patients or effective in reducing the severity of disease outcomes in home or clinical settings.

Understanding the relationship between the sociodemographic factors and the severity of dementia is critical for health policymakers, as well as clinicians, to develop adequate preventive and supportive strategies. Knowledge about the impact of informal caregiver’s role on severity of dementia is determinant to understand the kind and quality of support that patients should receive. The aim of our study is to explore the clinical dementia rating (CDR) score distribution for the patients with different family functional and sociodemographic issues, and to obtain knowledge on the important factors related to the severity of the dementia status, especially concerning the roles and effect of family members involved.

## 2. Materials and Methods

### 2.1. Study Design

A cross-sectional study was used to attain research objectives. The study was performed in a regional hospital in Central Taiwan. This hospital in Central Taiwan has 644 beds, with an average occupancy of 46.69% and an average of 1600 outpatient department visits per day in 2019. When the patient visits the neurology clinic for the first time, the psychological assessment using the Mini-Mental State Examination (MMSE) and CDR scales were administered by a clinical psychologist in a clinical psychological counseling room, following the order of the neurologist. 

A convenience sample of the patients diagnosed with dementia were recruited from the outpatient clinic during May 2018 to April 2019. The analysis for this study used the baseline data from a group of 318 patients (191 women and 127 men) who came to the clinic during May 2018 to April 2019, and who were diagnosed by physicians with the CDR scores ≧ 0.5. An anonymous analysis of the study data was used to confirm confidentiality.

### 2.2. Measurements

There are some commonly used tools for the preliminary diagnosis of dementia in the hospitals of Taiwan, such as MMSE and CDR. The Mini-Mental State Examination (MMSE) is a widely used tool to identify and rank cognitive impairment in elders [18]. It consists of a group of questions covering seven groups of cognitive functions, (1) orientation to time, (2) orientation to place, (3) registration of three words, (4) attention and calculation, (5) recall of three words, and (6) language and visual construction, with the maximum score of 30 points [19]. MMSE is found to be insensitive to the early signs of dementia, especially in highly educated individuals [20], so it is not considered in this study.

The clinical dementia rating (CDR) is an international scale established to indicate the presence and the severity phase of AD [21]. Its clinical protocol includes semi structured interviews, with the patient and informant to acquire information to evaluate the patient’s cognitive performance in six domains: memory, orientation, judgment and problem solving, community affairs, home and hobbies, and personal care. Each domain is rated on five levels of impairment: 0, 0.5, 1, 2, and 3 indicate none, questionable, mild, moderate, and severe dementia. The CDR is generally known in the clinical setting as a reliable and valid universal assessment tool for AD [22].

CDR scores, sociodemographic data (age ≤ 64 years; 65–74 years; 75–84 years and ≥85 years), sex, education level (illiterate; ≤6 years and ≥7 years), marriage status (unmarried; married and divorced/separated), and information regarding companions for clinic visits and housemates were obtained from interviews by a psychologist and from patient records.

Patients completed the CDR to evaluate cognitive and functional performance. The general CDR score of 0 indicates no dementia, and 0.5, 1, 2, and 3 indicate very mild, mild, moderate, and severe dementia. In this study, the description of CDR scores of the study samples were grouped as ≤1 and ≥2 for data analysis. 

### 2.3. Statistical Analysis

Frequency analyses were conducted to assess the distribution of CDR scores, and to classify the related factors of CDR scores among patients. To assess predictors of CDR scores levels, the Chi-Square test and binary logistic regressions were performed for inferential statistical analysis. The Stata software package (version 13.0; StataCorp., TX, USA 2013) [23] was used for statistical analyses.

### 2.4. Ethics Approval

The study protocol was approved by the Medical Ethics Committee of Jen-Ai Hospital (IRB no. 108-48). The institutional review board of the Medical Ethics Committee of Jen-Ai Hospital approved this study without requiring written informed consent form any study patients.

## 3. Results

### 3.1. Characteristics of the Study Sample

The study included 318 patients, mean age 78.9 ± 8.51 years. Of these, 129 (40.6%) had no more than 6 years education, 29.2% were illiterate, and most (96.5%) were married. There were 237 (74.5%) above 75 years of age. Furthermore, 6.9% of the patients went to the clinic by themselves. The others were accompanied by spouse (21.7%), sons or daughters-in-law (40.6%), daughters (23.9%) or others (6.9%). In total, 141 (44.3%) and 100 (31.4%) of the patients’ main caregivers were sons and spouses. After taking the CDR, 38.4% of their scores were equal or greater than 2.0. The samples’ basic sociodemographic and clinical characteristics are shown in Table 1.

### 3.2. Patient Characteristics, Family Status, and CDR Scores

Differences in the CDR score distribution among the dementia patients by the X^2^ tests are shown in Table 2. Since there were only 15 patients whose CDR scores equaled 3, we combined the cases of CDR scores ≥2 as the high scores group, and the others were the low scores group for the inferential statistics. Variables that reached a statistically significant difference include age (*p* = 0.001), educational level (*p* < 0.001), companions for clinic visits (*p* < 0.001), and main caregiver (*p* = 0.035). Comparing age and CDR scores, 27.8% of the patients younger than 65 had CDR scores ≥2, while 53.8% of those 85 or older were in this bracket, revealing that a high CDR score was related to higher age. Higher CDR scores were found in patients who were older or had less educational level. Among the patients with CDR scores ≥2, 57.0% were illiterate, compared to 33.3% for patients who received 1 to 6 years of elementary education and 27.1% for patients with 7 or more years of schooling. In the group of CDR scores for ≥2, only 9.1% went to the hospital by themselves, while 68.2% were accompanied to the hospital by relatives. Patients whose main caregiver were daughter and son with higher CDR scores (≥2) were 47.6% and 44.7%, respectively, and those whose main caregiver were spouses were 27.0%. Females have higher prevalence of CDR scores ≥2 than males (40.3% vs. 35.4%), but gender did not reach a statistically significant difference (*p* = 0.224).

### 3.3. Binary Logistic Regressions

Table 3 shows results from the binary logistic regression analyses, indicating that the difference of CDR scores among various age groups did not reach significant difference. This result shows that educational level and people accompanying the patient for a clinic visit are predictors for CDR score distribution. Patients who went for clinic visits by themselves had lower CDR scores than patients who were accompanied by family members. Results of patients who went to the clinic with others (OR = 18.871, 95% C.I. = 3.117 to 114.237, *p* = 0.001), accompanied by their spouse (OR = 10.783, 95% C.I. = 1.996 to 58.245, *p* = 0.006) and son/daughter in law (OR = 7.733, 95% C.I. = 1.545 to 38.699, *p* = 0.013), reached statistical significant difference.

CDR scores for patients whose main caregiver was their spouse (OR = 0.528) or daughter (OR = 0.952) were lower than those of the patients who lived by themselves. However, the distribution of CDR scores among different main caregivers did not reach significant difference.

## 4. Discussion

This study analyzes family support and the sociodemographic factors of dementia patients. We found that both the family member accompanying the patient to clinic visits and the patient’s educational level are significant issues relating to the severity of dementia. Since the person actually performing home care may be a more significant factor in the progression of the disease, this information would thus be more helpful for providing a direction for future research in planning effective care regimes for dementia patients.

Although some studies indicate that gender, age and education levels are important factors relating to the prevalence of dementia disease [24,25,26], our results indicated that there is no difference in CDR score distribution between male and female as shown in Table 2. Although the distribution of CDR scores in different age groups reached a significant level of difference (*p* = 0.001), the results of the binary logistic regression analysis revealed that age is not the major factor in the severity of dementia. The results imply that other physiological factors have more influence on dementia status. More research is needed to clarify the effect of age on dementia progression.

Our study finds that people with more years of formal education have lower CDR scores than the illiterate. Some researchers state that having more years of education enhances the brain’s ability to make efficient use of cognitive networks and supports the performance of cognitive tasks, which would help to prevent brain damage [27]. Those with more years of formal education tend to have occupations that are mentally more stimulating [28,29,30]. In addition, more years of formal education is associated with better socio-economic status [31], which may increase the likelihood of good nutrition and health care. People in the United States with fewer years of education are inclined to have more cardiovascular risk factors for Alzheimer’s, such as: (1) being more sedentary [32]; (2) having a higher prevalence of diabetes [33]; (3) cardiovascular disease [34]. The relationship between the effect of education and dementia in Taiwan needs further investigation.

Many studies discuss the burden of family members caring for patients with cognitive impairment, but only a few to clarify the care benefit of the family for dementia patients. It is found that more than 50% of caregivers have beneficial experiences such as enjoying closeness, sharing activities, sensing a mutual tie, spiritual and individual growth, improved confidence, and feelings of accomplishment and prosperity [35]. The present study found that patients whose main caregiver are sons have higher CDR scores than the others (OR = 2.664, 95% CI, 0.812–8.737, *p* = 0.106), and the results showed that descendent siblings of the household are typically likely to be thought to take the major responsibilities of taking care of their parents in Taiwanese ethos, but the results did not reach the statistical significant difference under the logistic regression analysis. In most Asian countries, sons are usually expected to be responsible for taking care of their parents, and twice as many parents live with a son as live with a daughter in Japanese families [36]. It is found that the Taiwanese public lacks knowledge about dementia and is less familiar with the symptoms of AD than Western countries [37]. Since families are the main informal caregivers for most patients with dementia, the relationship between the influence of the family function and the caring effects on dementia patients needs further investigation.

When a dementia patient visits an out-patient department (OPD), they may be disturbed by the boisterous environment in hospitals, complicated OPD routes and procedures. Family members who accompany persons with dementia to an OPD are important assistants, and in many cases, are the first to detect irregular changes in the patient and become the patient’s main caregivers. The main family caregivers can help doctors accurately diagnose dementia by having observed behavioral symptoms in daily life [38]. According to our study, the largest portion of patients (40.6%) was accompanied by their sons, and they had higher CDR scores than patients who came to the clinic alone, OR = 7.733 (95% CI, 1.545–38.699, *p* = 0.013). A similar result is observed that patients who went to the clinic with other relatives and spouses had higher ORs and reached a significant level of difference, OR = 18.871 (95% CI, 3.117–114.237, *p* = 0.001) and OR = 10.783 (95% CI, 1.996–58.245, *p* = 0.006). These results imply that offspring may provide better support for dementia patients, and the lowest OR were found in the patients who went to the clinic with their daughters, OR = 2.724 (95% CI, 0.544–13.813, *p* = 0.221), since daughters are usually not considered as a member of their original family after marriage under the traditional Chinese culture. They are also not expected to have the obligation of caring for their own parents. Due to the sharp reduction in Taiwan’s birth rate, daughters might need to accept the duty of care for their own nuclear family more than in the past [39]. Further investigation about the caring effect of different family companions for dementia patients may be helpful to design effective caring strategies, and the relationship between the severity of dementia and the effect of family companions for clinic needs further investigation. 

### Strengths and Limitations

We established the empirical finding that patients’ educational level and family function, such as impact of family involvement during medical visits, play an important role relating to the severity of dementia patients. There are some limitations of this study. The sample came from a single metropolitan-regional hospital in Central Taiwan, so the findings should not be regarded as the norm for all dementia patients in Taiwan. Our study is a cross-sectional study, so the actual effects of the factors relating to the severity of dementia need further exploration. Future research can consider the relationship between the caregiver at the initial assessment and the speed of cognitive decline in the future. 

## 5. Conclusions

This study determines that educational level and family function are important factors relating to the severity of dementia. To propose the continuous learning programs may enhance the stimulation of brain activity and prevent the occurrence or deterioration of dementia. It is found that social engagement has positive effect on general cognitive status [40]. To encourage the elderly dedicating social involvement may be helpful for dementia prevention. The companion of family member with the patient to clinic visits is significantly correlated to the CDR scores, and therefore, it is important to propose the effective propaganda program about the symptoms and care skill of dementia to the general citizen. Further investigation for the design of effective caring strategies should consider the effects of family companions on dementia patients.

## Figures and Tables

**Table 1:** Distribution of clinical dementia rating (CDR) scores and sociodemographic factors in the patients with dementia.

	N	%
CDR		
≤1.0	196	61.6
2.0	107	33.6
3.0	15	4.8
Sex		
Female	191	39.9
male	127	60.1
Age	Mean ± SD = 78.86 ± 8.51	
≤64	18	5.7
65–74	64	19.8
75–84	146	45.9
≥85	91	28.6
Marriage status		
Unmarried	7	1.6
Married	307	96.5
Divorced/separated	2	1.9
Educational level (years)		
Illiterate	93	29.2
≤6	129	40.6
≥7	96	30.2
Companion to clinic visit		
Self	22	6.9
Spouse	69	21.7
Son/daughter-in-law	129	40.6
Daughter	76	23.9
Others	22	6.9
Main caregiver		
Alone with/without caregiver	56	17.6
Spouse with/without daughter	100	31.4
Son	141	44.3
Daughter	21	6.6
Total	318	100.0

**Table 2:** The distribution of CDR scores in different factors.

	CDR Scores *p* Value N (%)			
	≤1.00	≥2.00	Total	
Gender				0.224
Female	114 (59.7)	77 (40.3)	191 (100)	
Male	82 (64.6)	45 (35.4)	127 (100)	
Age **				0.001
≤64	13 (72.2)	5 (27.8)	18 (100)	
65–74	48 (76.2)	15 (23.8)	63 (100)	
75–84	93 (63.7)	53 (36.3)	146 (100)	
≥85	42 (46.2)	49 (53.8)	91 (100)	
Educational level (years) **				<0.001
Illiterate	40 (43.0)	53 (57.0)	93 (100)	
≤6	86 (66.7)	43 (33.3)	129 (100)	
≥7	70 (72.9)	26 (27.1)	96 (100)	
Companion to clinic visit ***				<0.001
Self	20 (90.9)	2 (9.1)	22 (100)	
Spouse	45 (65.2)	24 (34.8)	69 (100)	
Son/daughter-in-law	67 (51.9)	62 (48.1)	129 (100)	
Daughter	57 (75.0)	19 (25.0)	76 (100)	
Others	7 (31.8)	15 (68.2)	22 (100)	
Main caregiver *				0.035
Alone with/without caregiver	34 (60.7)	22 (39.3)	56 (100)	
Spouse	73 (73.0)	27 (27.0)	100 (100)	
Daughter	11 (52.4)	10 (47.6)	21 (100)	
Son	78 (55.3)	63 (44.7)	141 (100)	

*: *p* < 0.05; **: *p* < 0.01; ***: *p* < 0.001.

**Table 3:** Logistic regression analysis of CDR scores in different factors.

	OR	95% C.I.	*p* Value
Age			
≤64	Reference		
65–74	0.873	0.255–2.989	0.829
75–84	1.084	0.336–3.493	0.893
≥85	2.289	0.696–7.522	0.173
Educational level			
Illiterate	2.725	1.337–5.553	0.006
≤6	1.193	0.630–2.261	0.588
≥7	Reference		
Companion to clinic visit			
Self	Reference		
Spouse	10.783	1.996–58.245	0.006
Son/daughter-in-law	7.733	1.545–38.699	0.013
Daughter	2.742	0.544–13.813	0.221
Others	18.871	3.117–114.237	0.001
Main caregiver			
Alone with/without caregiver	Reference		
Spouse	0.528	0.218–1.276	0.156
Son	2.664	0.812–8.737	0.106
Daughter	0.952	0.457–1.980	0.895
			R^2^ = 0.231

CDR scores for patients whose main caregiver was their spouse (OR = 0.528) or daughter (OR = 0.952) were lower than those of the patients who lived by themselves. However, the distribution of CDR scores among different main caregivers did not reach significant difference.

## Data Availability

The data is available by contacting the corresponding author.
